# Interventional Treatment Is an Effective Approach to Relieve Symptoms in Patients With Malignant Tracheoesophageal Fistula

**DOI:** 10.1002/cnr2.70312

**Published:** 2025-08-10

**Authors:** Pei Huang, Jinghua Cui, Zhenbang Lie, Jing Li

**Affiliations:** ^1^ Department of Oncology Dongguan Kanghua Hospital Dongguan Guangdong China; ^2^ Department of Pulmonary and Critical Care Medicine Guangdong Provincial People's Hospital (Guangdong Academy of Medical Sciences), Southern Medical University Guangzhou Guangdong China; ^3^ Department of Cardiology Dongguan People's Hospital Dongguan Guangdong China; ^4^ Department of Pulmonary and Critical Care Medicine The First People's Hospital of Yunnan Province, the Affiliated Hospital of Kunming University of Science and Technology Kunming Yunnan China

**Keywords:** airway intervention, esophageal cancer, esophageal intervention, overall survival, tracheoesophageal fistula

## Abstract

**Background:**

A malignant tracheoesophageal fistula (mTEF) is a complication of primary tumor growth or the recurrence of esophageal tumors or lung carcinoma. Patients with mTEF have lower survival and quality of life than those who do not develop this complication. Esophageal cancer (EC), a common gastrointestinal malignancy, ranks among the world's leading causes of cancer‐related death. The low survival rate in patients with EC is attributed to malnutrition, repeated aspiration, and severe infection; the mean survival duration is 2–4 months after diagnosis. This study investigated the clinical characteristics of patients with EC complicated with mTEF and the efficacy of various treatment regimens.

**Methods:**

This study was a retrospective analysis of the clinical data of 51 patients with EC complicated with mTEF hospitalized at Guangdong Provincial People's Hospital from February 2007 to May 2021. Patients were divided into three groups according to their treatment regimen: a traditional medical (TM) treatment group, an esophageal intervention (EI) treatment group, and an airway intervention (AI) treatment group.

**Results:**

Of the 51 patients, 22 received TM treatment, 13 received AI, and 16 received EI. The overall median survival duration was 87 days (TM group, 42 days; AI group, 108 days; EI group, 104 days) and the overall mean survival duration was 130.1 days (TM group, 88.1 days; AI group, 153.5 days; EI group, 166.1 days). Cox regression analysis revealed that the treatment regimen was an independent predictive risk factor for increased survival 1 month after treatment in patients with EC complicated with mTEF, and most symptoms were relieved in the EI and AI groups.

**Conclusions:**

Interventional treatment of the esophagus and airway in patients with EC complicated with mTEF is an effective approach to improve symptoms and increase short‐term survival.

## 1 Introduction

Malignant tumor‐related tracheoesophageal fistula (mTEF) is a pathological connection between the esophagus and airway due to primary tumor growth or the recurrence of esophageal or lung carcinoma [1, 2]. It can occur secondary to radiation or chemotherapy, resulting in tumor dehiscence, poor quality of life (QoL), and low survival. Approximately 70% of mTEF are associated with esophageal cancer (EC), 20% with lung cancer, and a small proportion with other tumors, such as malignant mediastinal lymph node lesions, thyroid cancer, and laryngeal cancer [3].

Approximately 10% of patients with EC develop mTEF, trapping them in a vicious cycle of choking during eating, repeated pulmonary infections, and cachexia [4]. In patients with EC complicated by mTEF, frequent airway aspiration, malnutrition, and severe infections can cause rapid deterioration. Their QoL scores are on average > 40% lower than those of tumor patients without fistulas, with a 1‐year survival rate < 15% and most surviving only 2–4 months after diagnosis [5, 6, 7]. There is an urgent need for more effective interventions to break through this therapeutic bottleneck.

Therapeutic strategies for mTEF have undergone continuous development, including surgical resection/fistula repair, medical supportive care, and interventional treatment. Surgery is the first‐line treatment for EC patients with mTEF, but it has limited efficacy. The reported surgical closure rate was only 1.9% (14/733) for a bronchial fistula occurring within 14 days after pneumonectomy; furthermore, most patients are not suitable candidates for surgery because of severe infection and poor general condition [8]. Traditional medical support care also has limited effects, and it provides no obvious relief from short‐term symptoms. By contrast, interventional treatment has emerged as a core development direction in mTEF treatment due to its minimally invasive nature, target specificity, and rapid symptom control. It enables enteral nutrition, direct fistula closure, and lower pulmonary infection risk, relieving symptoms quickly and reducing surgical needs. Guided by endoscopy or imaging, these techniques enable precise placement of fistula occluders and establishment of enteral nutrition pathways. Avoiding thoracotomy trauma, these techniques reduce pulmonary infection incidence by 60%–70% and relieve choking symptoms in > 90% of patients within 48 h postoperatively [9, 10]. More groundbreaking is the development of emerging biodegradable occlusive materials, which are rewriting the long‐term complication challenges of traditional metal stents and offering new pathways for biological fistula closure [11].

However, significant clinical evidence gaps exist in interventional treatment for EC with mTEF, including lacking standardized treatment selection guidelines, unclear long‐term survival impacts, and particularly undefined mechanisms on whether it can create opportunity windows for subsequent tumor chemoradiotherapy [12]. Through retrospective analysis, this study systematically compares the efficacy of esophageal stenting (EI), airway stenting (AI), and traditional therapy (TM) for the first time, aiming to establish evidence‐based personalized treatment strategies for mTEF and provide critical clinical evidence to address this “dead end in cancer treatment.”

## 2 Materials and Methods

### 2.1 Study Design and Ethics

This study is a single‐center retrospective study, which has been approved by the Research Ethics Committee of Guangdong Provincial People's Hospital (approval no. KY‐Q‐2022‐079‐01). Due to the retrospective nature of the study, the ethics committee waived the requirement for informed consent.

### 2.2 Patients

This study included patients hospitalized at Guangdong Provincial People's Hospital between February 2007 and May 2021, aged 20–80 years, with histologically confirmed EC and diagnosed with esophagotracheal fistula via upper gastrointestinal radiography, fibroesophagoscopy, bronchoscopy, or chest computed tomography. Patients were ineligible for surgery due to malnutrition, high disease severity, poor general condition, high surgical risk, or refusal of treatment. Additionally, patients who underwent open surgical repair, thoracoscopic intervention, or had unavailable survival data were excluded from this study.

Subsequently, the enrolled patients were divided into three groups according to the treatment received: traditional medical treatment (TM) group, esophageal interventional therapy (EI) group, and airway interventional therapy (AI) group. Patients in the TM group received noninvasive treatments such as anti‐infection therapy, thoracic drainage, intravenous nutritional support, nasogastric feeding, and gastrostomy. Patients with stent implantation were classified into esophageal stent group and airway stent group according to stent type. Patients in the EI group underwent covered esophageal stent implantation, while patients in the AI group received direct occlusion device implantation under fiberoptic bronchoscopy guidance, such as atrial septal occluders or airway stents.

**FIGURE 1:**
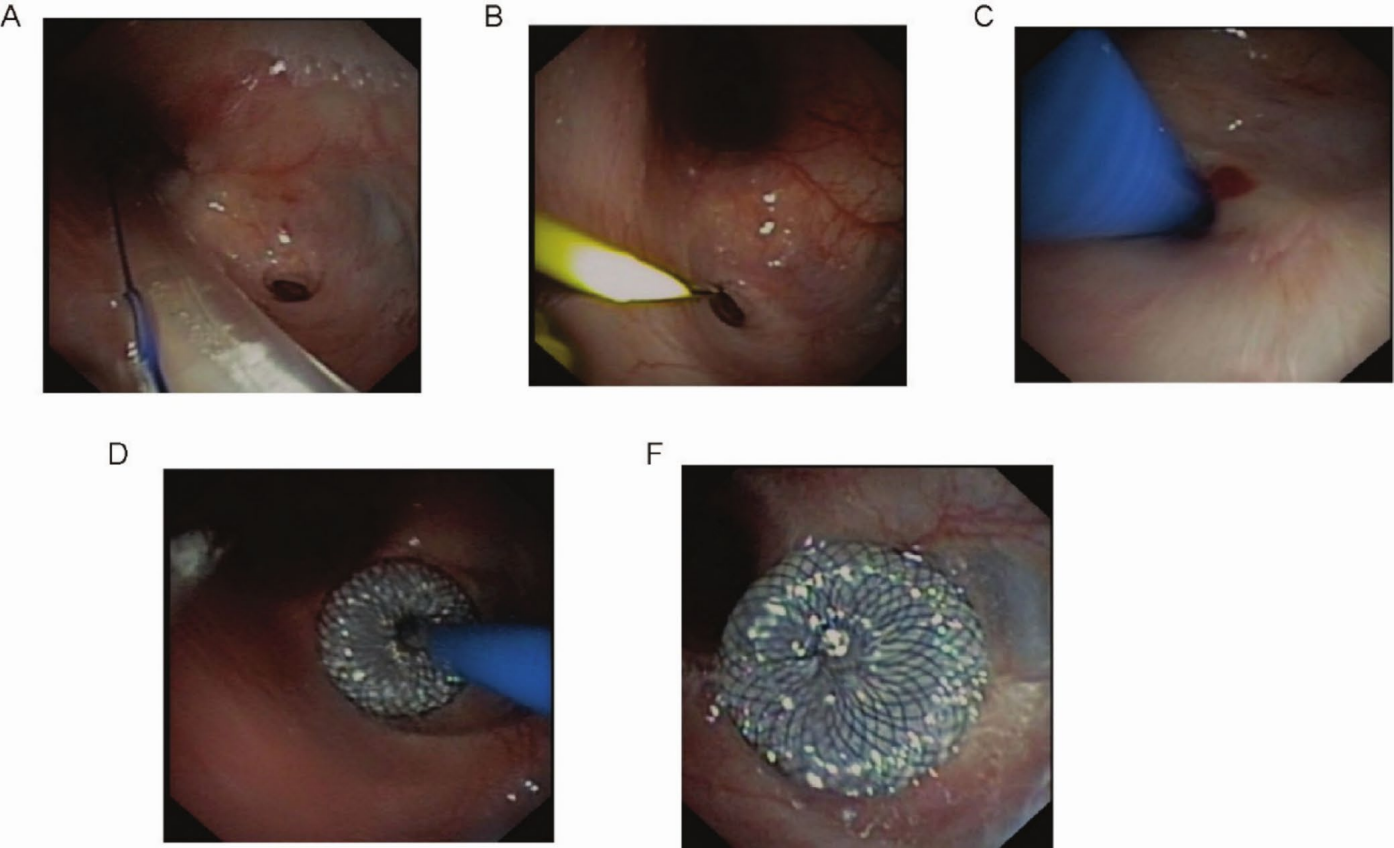
Interventional procedure. (A) A fistula is visualized via gastroscopy. (B) A guidewire is inserted via bronchoscopy. (C) Guidewire‐guided sheath insertion. (D) Release the occluder. (E) A fistula occluded by an occluder.

For AI procedures, the selection of occlusion devices or stents was based on lesion characteristics: self‐expanding metallic stents (e.g., nitinol) were preferred for complex airway stenosis with elastic recoil, while silicone stents were used for benign strictures requiring easier removability. Modified atrial septal occluders were selected for tracheobronchial fistulas with defect diameters ≤ 15 mm, matched to fistula size via pre‐procedural imaging. For EI, covered esophageal stents were chosen based on lesion location (proximal/mid/distal esophagus) and severity. AI procedures were performed under general anesthesia with continuous vital sign monitoring, involving bronchoscopic visualization of airway lesions (e.g., stenosis or fistulas), followed by fluoroscopically guided deployment of airway stents or modified atrial septal occluders to restore patency or seal defects. EI involved conscious sedation or general anesthesia, fluoroscopic navigation for esophageal stent placement, with pre‐procedural endoscopic measurement of lesions and deployment of covered stents to prevent leakage, followed by post‐implantation assessment of patency and complications. The specific interventional procedure can be seen in Figure 1.

### 2.3 Data Acquisition

The baseline characteristics of the patients (age, sex, initial tumor staging, fistula location, fistula cause, and therapeutic effects) were recorded. Initial tumor staging refers to the tumor stage at the time of initial diagnosis. The causes of fistula formation include tumor invasion, residual tumor progression after radical surgery, and tumor regression during radiotherapy and chemotherapy. Currently, there is no standardized criterion for evaluating the treatment response of airway fistulas. Wang et al. established a standard evaluation to determine the efficacy of stent fistula closure according to experience from multiple centers in China [13]. Patients in complete remission (CR) exhibit a sealing of the fistula and complete relief of clinical symptoms (such as choking while drinking, fever, etc.) for one month; patients in clinical complete remission (cCR) have a sealed fistula that is completely occluded by stents along with complete relief of clinical symptoms for one month; patients in partial remission (PR) exhibit a fistula that is not closed and is partially occluded by a stent, along with partially relieved clinical symptoms; and patients not in remission (NR) are those in whom the fistula is not closed and not occluded by a stent, and the clinical symptoms are not relieved. The overall response rate (ORR) is the sum of CR, cCR, and PR. The treatment outcomes of this study were evaluated with reference to the above criteria. Tumor staging was determined according to the 8th edition of the American Joint Committee on Cancer Staging Manual [14].

### 2.4 Patient Follow‐Up

All patients were followed up through outpatient data, inpatient data, and telephone calls. Follow‐ups occurred 1 month after discharge to assess the therapeutic effects and every 2 months thereafter or as needed according to the patient's symptoms. The referring doctor conducted long‐term follow‐ups via telephone for patients who were unable to routinely visit our clinic.

### 2.5 Statistical Analysis

Data are presented as the mean ± standard deviation (for normally distributed continuous data), or the number (%) (for categorical data), or the median (interquartile range, IQR) (for non‐normally distributed continuous data). For comparisons of continuous data, the unpaired *t*‐test was used for normally distributed variables, while the Mann–Whitney *U* test was applied for non‐normally distributed variables. For categorical data, Pearson's chi‐square test was used when sample sizes were sufficient, and Fisher's exact test was employed for small sample sizes or sparse data. Survival data were analyzed using the Kaplan–Meier method with log‐rank tests for group comparisons, and the Cox proportional hazards regression model was used for multivariate analysis of survival factors. For chi‐square comparisons across three groups, post hoc pairwise comparisons were adjusted using the Bonferroni correction, with statistical significance set at a corrected threshold of *p* < 0.016 (0.05/3). For all other tests, statistical significance was defined as *p* < 0.05. All statistical analyses were performed using IBM SPSS Statistics for Windows, version 20.0 (IBM Corporation, Armonk, NY, USA).

## 3 Results

### 3.1 Demographics

Of the 51 patients with EC complicated with mTEF, 46 were male and five were female; the mean age was 58.2 ± 1.1 years. The patients were divided into three groups: TM group (22 patients with an average age of 58.6 ± 1.8 years), an EI group (16 patients with an average age of 57.5 + 1.9 years), and an AI group (13 patients with an average age of 58 ± 2.2 years).

The main causes of EC complicated with mTEF were tumor invasion, radiotherapy, chemotherapy, and radical surgery. Direct invasion of EC accounted for 43.1% of the cases (22/51); radiotherapy accounted for 23.5% of the cases (12/51); radical surgery accounted for 29.5% of the cases (15/51); and chemotherapy accounted for 3.9% of the cases (2/51) (Table 1).

**TABLE 1:** Patient demographic data.

Variable	Patients, no. (%)		
	AI	EI	TM
Sex			
Male	11 (21.6)	15 (29.4)	21 (41.1)
Female	2 (3.9)	1 (2.0)	1 (2.0)
Age, years			
≥ 55	10 (19.6)	10 (19.6)	14 (27.5)
< 55	3 (5.9)	6 (11.8)	8 (15.7)
Fistula cause			
Tumor invasion	3 (5.9)	9 (17.6)	10 (19.6)
Radical surgery	6 (11.8)	1 (2.0)	8 (15.7)
Radiotherapy	3 (5.9)	5 (9.8)	4 (7.8)
Chemotherapy	1 (2.0)	1 (2.0)	0 (2.0)
Fistula location in airway			
Trachea	10 (19.6)	7 (13.7)	18 (35.3)
Main bronchus	3 (5.9)	7 (13.7)	3 (5.9)
Bronchus	0 (0)	2 (4.0)	1 (2.0)
Initial tumor staging			
I + II	3 (5.9)	1 (2.0)	4 (7.8)
III	5 (9.8)	10 (19.6)	13 (25.5)
IV	5 (9.8)	5 (9.8)	5 (9.8)

Abbreviations: AI, airway interventional therapy group; EI, esophageal interventional therapy group; TM, traditional medical treatment group.

### 3.2 Therapeutic Effects

We did not identify a significant influence of sex, age, fistula cause, fistula location, or tumor stage on therapeutic effect, but we did observe significant differences among the different treatment groups (*p* = 0.015), with better symptom improvement in the AI group than the TM group. By contrast, the results did not indicate a significant difference in symptom improvement between the EI and TM groups (Table 2).

**TABLE 2:** Therapeutic effects among the treatment groups.

Variable	ORR (*n* = 43)	NR (*n* = 19)	*χ* ^2^	*p*
Sex				
Male	28	18	0.706	0.639
Female	4	1		
Age				
≥ 55 years	22	11	0.615	0.547
< 55 years	10	8		
Fistula cause				
Tumor invasion	12	10	1.570	0.666
Radical surgery	11	4		
Radiotherapy	8	4		
Chemotherapy	1	1		
Fistula location in airway				
Trachea	24	11	5.569	0.062
Main bronchus	5	8		
Bronchus	3	0		
Treatment group				
AI	11^a^	2^a^	8.176	0.015*
EI	12^a,b^	4^a,b^		
TM	9^b^	13^b^		
Tumor stage				
I + II	7	1	4.535	0.104
III	18	10		
IV	7	8		

*Note:* ORR = CR + cCR + PR. a and b are used for intergroup comparison labeling. Each superscript letter denotes a subset of groups whose proportions do not differ significantly (chi‐square test, *p* > 0.05).

Abbreviations: AI, airway interventional therapy group; cCR, clinical complete remission; CR, complete remission; EI, esophageal interventional therapy group; NR, not in remission. PR, partial remission; TM: traditional medical treatment group.

* Denotes the comparison between the AI group and the TM group, with a *p*‐value of 0.015.

### 3.3 Survival Analysis

#### 3.3.1 Survival

**FIGURE 2:**
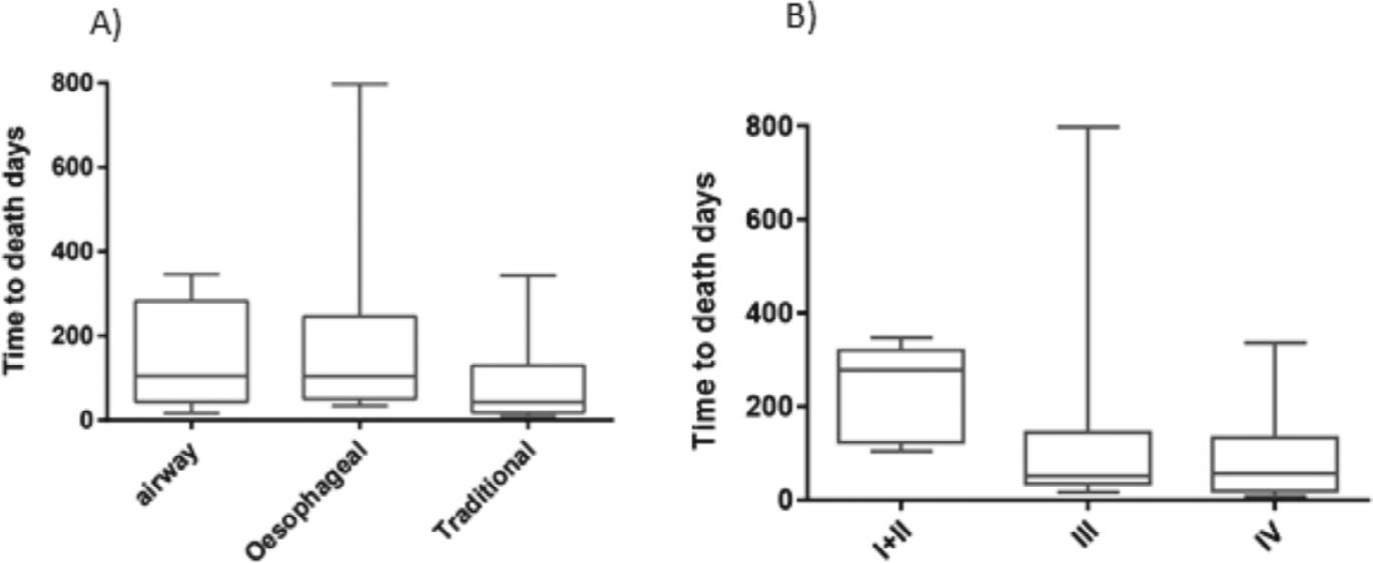
Survival analysis with independent factors. Box plots showing the relationship between (A) survival time and treatment and (B) survival time and tumor stage. The boxes represent the median and interquartile range (IQR), and the whiskers represent ±1.5 IQR.

The median overall survival (OS) duration was 87 days (TM group, 42 days; AI group, 108 days; EI group, 104 days) and the mean OS duration was 130.1 days (TM group, 88.1 days; AI group, 153.5 days; EI group, 166.1 days). The survival duration was significantly shorter in the TM group than in the AI and EI groups (*p* = 0.032 and *p* = 0.030, respectively; Table 3). Figure 2 presents the survival duration according to the treatment regimen and fistula stage.

**TABLE 3:** Survival analysis.

Variable	*n*	Mean survival	Median survival	*p*
Sex				
Male	46	128.9	87.0	0.82
Female	5	143.0	150.0	
Age				
≥ 55 years	33	131.5	91.5	0.65
< 55 years	18	145.3	76.0	
Fistula cause				
Invasion of fistula	22	95.1	76.0	0.074
Radical of fistula	15	230.2	222.5	
Radiotherapy	11	99.4	49	
Chemotherapy	3	32	32	
Fistula location				
Trachea	35	138.9	91.5	0.27
Main bronchus	13	88.4	49	
Bronchus	3	211	196	
Treatment				
AI	13	153.5	108.0	0.041
EI	16	166.1	104.0	
TM	22	88.1	42	
Tumor stage				
I + II	8	244.4	276.0	0.012
III	28	120.8	52.5	
IV	15	93.9	56	
Total	51	130.1	87.0	

*Note:* AI vs. TM, *p* = 0.032; EI vs. TM, *p* = 0.030; AI vs. EI, *p* = 0.917.

Abbreviations: AI, airway interventional therapy group; EI, esophageal interventional therapy group; TM, traditional medical treatment group.

#### 3.3.2 Survival at 1 Month, 6 Months, and Overall Survival (OS)

**FIGURE 3:**
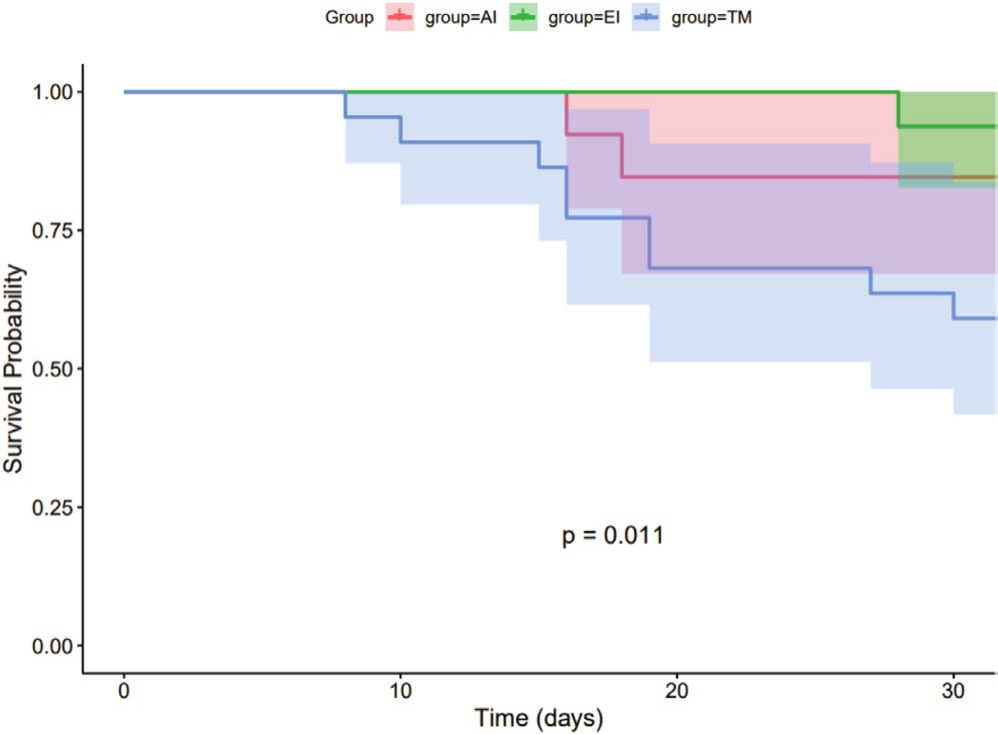
Kaplan–Meier analysis of 1‐month survival in patients with EC with mTEF.

**FIGURE 4:**
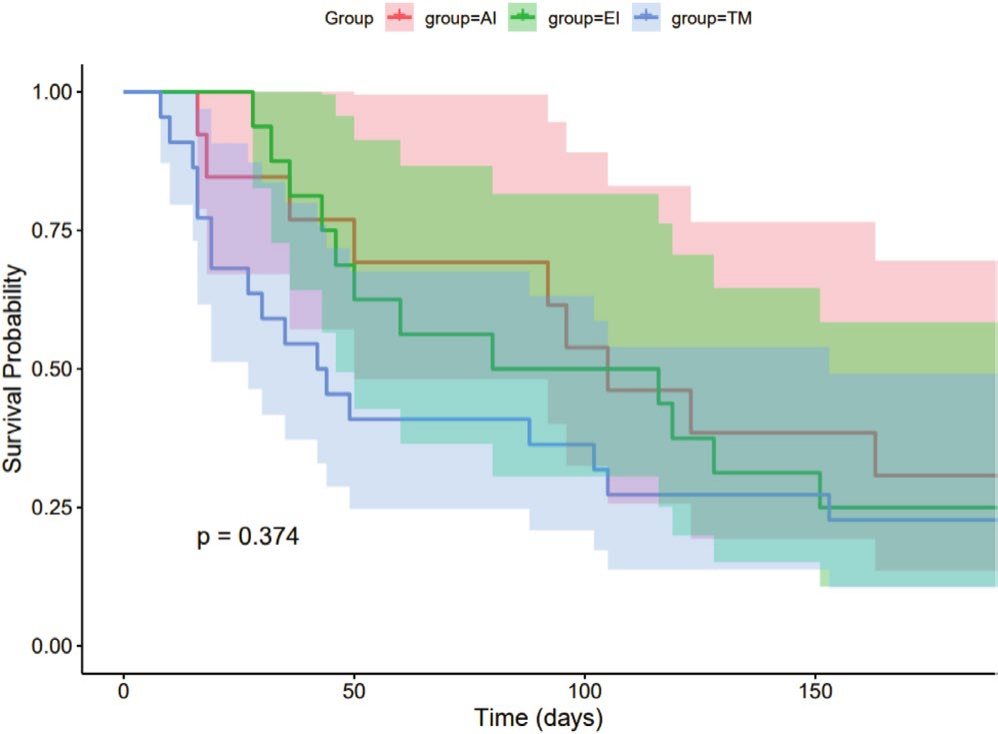
Kaplan–Meier analysis of 6‐month survival in patients with EC with mTEF.

**FIGURE 5:**
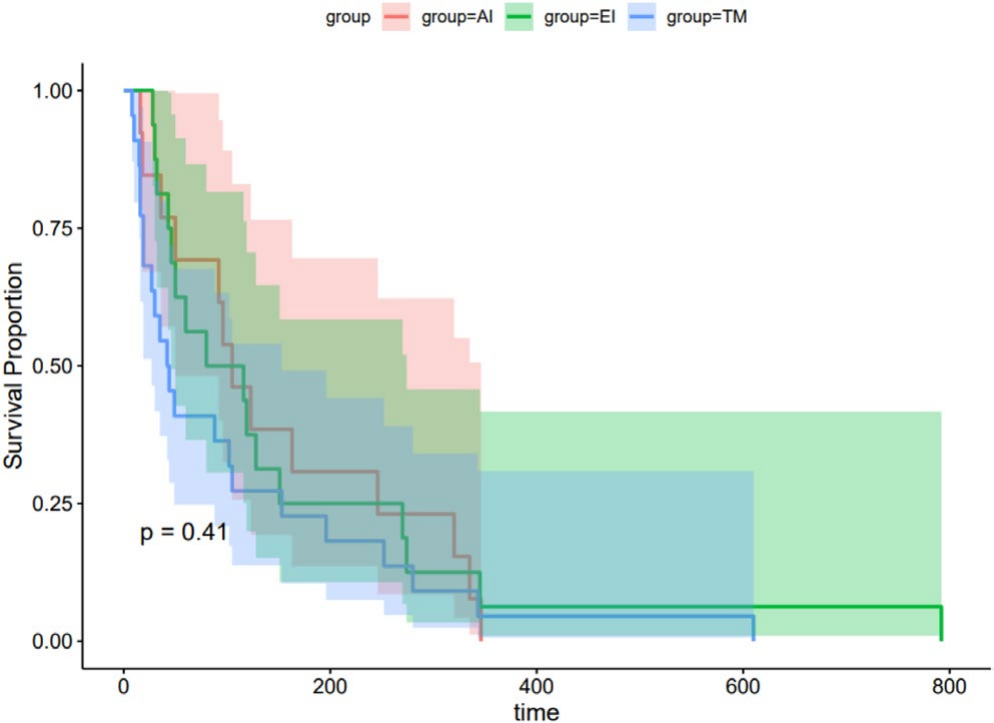
Kaplan–Meier analysis of OS in patients with EC with mTEF.

The survival curves created using the Kaplan–Meier method showed that 1‐month survival was higher in the AI and EI groups than in the TM group (*p* = 0.011) (Figure 3). However, survival at 6 months (*p* = 0.374, Figure 4) and OS (*p* = 0.41, Figure 5) did not differ between groups.

Univariate Cox analysis showed that the treatment regimen was an independent predictor of 1‐month survival but not 6‐month survival (Table 4). However, the analysis indicated that fistula cause and tumor stage were independent predictors of 6‐month survival.

**TABLE 4:** Cox analysis of survival at 6 months.

Variable	Low‐risk groups vs. high‐risk	Univariate analysis			Multivariate analysis		
		Hazard ratio	*p*	95% CI	Hazard ratio	*p*	95% CI
Sex	Female vs. male	2.537	0.201	0.609–10.567			
Age	≥ 55 vs. < 55 years	0.967	0.922	0.494–1.894			
Treatment	EI vs. TM	0.604	0.222	0.269–1.357			
	AI vs. TM	0.669	0.288	0.319–1.404			
Fistula cause	Radical of fistula vs. invasion of fistula	0.288	0.008	0.114–0.726	0.328	0.018	0.130–0.829
	Radiotherapy vs. invasion of fistula	0.871	0.733	0.395–1.923	0.838	0.663	0.379–1.852
	Invasion of fistula vs. chemotherapy	2.895	0.095	0.831–10.089	2.479	0.154	0.710–8.651
Fistula location	Main bronchus + bronchus vs. trachea	0.861	0.663	0.440–1.686			
Tumor stage	I + II + III vs. IV	0.152	0.010	0.036–0.634	0.182	0.020	0.043–0.766

Abbreviations: AI, airway interventional therapy group; EI, esophageal interventional therapy group; TM, traditional medical treatment group.

## 4 Discussion

EC is a common malignant tumor of the digestive tract. Tumor invasion, radiotherapy, chemotherapy, and radical surgery in advanced EC can often lead to an mTEF [15]; approximately 60% of which are secondary to chemoradiotherapy [1, 2]. Due to its propensity to cause life‐threatening secondary complications such as severe malnutrition, aspiration pneumonia, and massive hemorrhage, most patients die within 3–4 months of diagnosis, with a median survival period of only 1–6 weeks. Despite the rapid advancements in cancer treatment and supportive care, the incidence of EC complicated with mTEF has been increasing annually. For example, a study by Manini et al. involving 1943 EC patients in the 1970s reported an mTEF incidence of only 4.94% [16]. Since 2010, the incidence of EC with mTEF has risen to 8%–10% [17].

The treatment modalities for mTEF include surgical and nonsurgical approaches. Surgery remains the preferred treatment for mTEF, but the reported closure rate of early bronchial fistulas within 14 days after pneumonectomy is only 1.9% (14/733). Moreover, most patients are ineligible for surgery due to severe infection and malnutrition [8]. Nonsurgical treatments encompass tumor medical therapy, chemoradiotherapy, and interventional therapy. Tumor medical therapy (TM) has limited value in mTEF patients, with unclear efficacy in symptom relief [18].

In the treatment of TEF, radiotherapy and chemotherapy are perhaps the most controversial. At the beginning of this century, some scholars still advocated that radiotherapy and chemotherapy should be included in mTEF treatment protocols [2], arguing that there was no evidence that they would exacerbate mTEF progression and claiming they could improve patient survival. Although radiotherapy and chemotherapy are not formally recommended as standard treatment for TEF, in clinical practice, certain antitumor therapies are still administered to patients with good general condition and no absolute contraindications [19].

The goal of interventional therapy is to create an occlusion between the airway and esophagus, thereby enabling enteral nutrition and reducing bronchial contamination, so as to rapidly relieve symptoms and avoid the need for surgery [20, 21]. Current interventional treatments mainly include two types. One is biological adhesives: in some previous studies, endoscopic injection of “biological glues” such as fibrin glue has achieved efficacy for smaller fistulas (diameter < 5 mm). However, for larger fistulas, the effectiveness of such adhesives is limited, and they may damage the endoscope if they flow into the operating channel. The other is mesenchymal stem cell transplantation, which also holds promise for TEF repair but remains in the exploratory stage [22].

Another interventional treatment is stent implantation. Placing airway or esophageal stents has become the most commonly used supportive treatment for mTEF. Currently, there are two main interventional occlusion methods: one is to occlude the fistula by placing an esophageal stent via gastroscopy, and the other is to place an occluder or airway stent via bronchoscopy. If the size and location of the fistula are suitable, occluders commonly used in cardiology, such as ventricular septal defect (VSD) or atrial septal defect (ASD) occluders, are also an option [23]. In this study, the interventional treatments used included conventional esophageal stents and airway stents, and a small number of patients also received ventricular septal defect occluders.

This approach of placing stents through natural lumens to occlude mTEF is minimally invasive. It is reported that the short‐term aspiration symptom relief rate can reach 75%–90% [9, 10]. For example, Fruchter et al. treated 31 mTEF patients with atrial septal defect occluders and found that 30 patients experienced immediate symptom relief after treatment, making it suitable for all emergency cases and patients requiring long‐term treatment [24]. Our study results also showed that AI and EI can fully relieve the symptoms of advanced EC patients with mTEF within 1 month after treatment, with ORR rates of 84.6% and 75%, respectively. In terms of symptom improvement, our results are basically consistent with previous reports.

Meanwhile, these two types of stents also have their respective advantages and disadvantages. Compared with esophageal stents, airway stents can not only treat mTEF but also prevent airway stenosis. Additionally, although esophageal stents can better address the problem of dysphagia, their displacement may lead to life‐threatening risks of airway stenosis and bleeding; this may also account for the survival differences between the two interventional methods.

Balazs et al. conducted a retrospective survey of 264 EC patients with mTEF and found that the survival time of patients who received esophageal interventional therapy (EI) was significantly longer than that of patients who received nutritional support (3.4 vs. 1.8 months) [17]. Furthermore, Herth et al. performed a prospective study on 112 mTEF patients who underwent airway and/or esophageal stent implantation: 65 patients received airway stents, 37 received esophageal stents, and 10 received combined airway‐esophageal stents [25]. The study showed an average OS of 236.6 days (219.1 days in the airway stent group, 262.8 days in the esophageal stent group, and 252.9 days in the combined stent group), and the QoL of all three groups significantly improved after treatment.

The results of our study also showed that patients' symptoms significantly improved after interventional therapy. The OS time was 130.1 days (TM group, 42 days; AI group, 108 days; EI group, 104 days). The difference in average survival time between our study and Herth's study may stem from the following reasons: the average age of patients in our study was 58 years, while that in Herth's prospective study was 52 years. Another explanation is that Herth's study was prospective, with regular follow‐ups and more symptomatic treatments after interventional therapy; in contrast, our study was retrospective, and most patients did not receive regular follow‐ups after interventional therapy, leading to significant differences in survival time.

Since Herth's study was published, many studies have explored the safety and efficacy of AI and EI in the treatment of mTEF; but few have compared the efficacy of AI and EI.

This study is consistent with previous findings, showing that patients treated with AI or EI had better survival rates than those treated with TM. One possible explanation is that patients in the AI and EI groups had better general conditions than those in the TM group, likely because interventional therapy enables enteral nutrition and reduces bronchial contamination. Tumor stage was also associated with survival, suggesting that AI may prolong survival in patients with Stage I, II, and III EC.

Kaplan–Meier analysis showed that the 1‐month survival rate of patients in the AI and EI groups was higher than that in the TM group, but the analysis did not show significant differences in 6‐month survival rates and OS between groups (Figure 3). This finding suggests that EI and AI can reduce the short‐term mortality of mTEF patients, but may not significantly improve long‐term mortality and OS. One possible reason is that interventional therapy improves early survival by creating an occlusion between the airway and esophagus to enable enteral nutrition and reduce aspiration. However, in addition to tumor progression, these patients also face malnutrition, repeated airway aspiration, and episodes of pneumonia, which may exacerbate their condition. Therefore, compared with TM treatment, interventional therapy appears to confer higher early survival rates in EC patients with mTEF.

This study has several limitations. First, it is a single‐center study, so only a limited number of patients could be included. Second, the follow‐up period was relatively short. Therefore, prospective randomized controlled trials with longer follow‐up periods and larger sample sizes are needed to validate these results.

## 5 Conclusion

In summary, this study confirms that AI and EI can serve as effective treatment modalities for EC patients with mTEF. These interventions not only reduce short‐term mortality by occluding fistulas and restoring enteral nutrition, but also enable AI to rapidly improve symptoms such as dyspnea within 1 month, thereby enhancing QoL. The findings suggest that clinicians should actively incorporate AI and EI into first‐line palliative treatment strategies for EC with mTEF, especially for elderly patients and other critically ill individuals unable to tolerate surgery. The minimally invasive nature and low complication rate of these interventions provide a rationale for their promotion in primary healthcare settings. Future research should further optimize treatment protocols through multicenter studies.

## Author Contributions

**Pei Huang:** acquisition of data and drafting the initial manuscript. **Jinghua Cui:** conception and design of the study. **Zhenbang Lie:** analysis and interpretation of data. **Jing Li:** critical revision of the manuscript for important intellectual content and final editing. All authors contributed to the study, approved the final version of the manuscript, and agree to be accountable for all aspects of the work.

## Conflicts of Interest

The authors declare no conflicts of interest.

## Data Availability

The data that support the findings of this study are available on request from the corresponding author. The data are not publicly available due to privacy or ethical restrictions.

## References

[1] H. S. Kim , D. Khemasuwan , J. Diaz‐Mendoza , and A. C. Mehta , “Management of Tracheo‐Oesophageal Fistula in Adults,” European Respiratory Review 29, no. 158 (2020): 200094, 10.1183/16000617.0094-2020. 33153989 PMC9488631

[2] F. M. Shamji and R. Inculet , “Management of Malignant Tracheoesophageal Fistula,” Thoracic Surgery Clinics 28, no. 3 (2018): 393–402, 10.1016/j.thorsurg.2018.04.007. 30054077

[3] H. Wang , M. Tao , N. Zhang , et al., “Airway Covered Metallic Stent Based on Different Fistula Location and Size in Malignant Tracheoesophageal Fistula,” American Journal of the Medical Sciences 350 (2015): 364–368, 10.1097/MAJ.0000000000000565. 26422803

[4] M. F. Reed and D. J. Mathisen , “Tracheoesophageal Fistula,” Chest Surgery Clinics of North America 13, no. 2 (2003): 271–289, 10.1016/s1052-3359(03)00030-9. 12755313

[5] B. L. Bick , L. M. Song , N. S. Buttar , et al., “Stent‐Associated Esophagorespiratory Fistulas: Incidence and Risk Factors,” Gastrointestinal Endoscopy 77, no. 2 (2013): 181–189, 10.1016/j.gie.2012.10.004. 23245798

[6] I. Ahmad , K. S. Chufal , R. Bajpai , et al., “Malignant Esophagorespiratory Fistulas: A Comparative Effectiveness Survival Analysis,” BMJ Supportive & Palliative Care 14 (2020): e1700–e1703. 10.1136/bmjspcare-2020-002370 32826266

[7] J. K. Grass , N. Küsters , F. L. von Döhren , et al., “Management of Esophageal Cancer‐Associated Respiratory‐Digestive Tract Fistulas,” Cancers 14, no. 5 (2022): 1220, 10.3390/cancers14051220. 35267527 PMC8909259

[8] A. Mazzella , A. Pardolesi , P. Maisonneuve , et al., “Bronchopleural Fistula After Pneumonectomy: Risk Factors and Management, Focusing on Open‐Window Thoracostomy,” Seminars in Thoracic and Cardiovascular Surgery 30 (2018): 104–113, 10.1053/j.semtcvs.2017.10.003. 29109057

[9] M. Koch , A. Vasconcelos Craveiro , K. Mantsopoulos , M. Sievert , A. O. Gostian , and H. Iro , “Analysis of Surgical Treatment Strategy and Outcome Factors in Persistent Tracheoesophageal Fistula: A Critical Analysis of Own Cases and Review of the Literature,” European Review for Medical and Pharmacological Sciences 26, no. 1 (2022): 257–269, 10.26355/eurrev_202201_27776. 35049003

[10] W. A. Ross , F. Alkassab , P. M. Lynch , et al., “Evolving Role of Self‐Expanding Metal Stents in the Treatment of Malignant Dysphagia and Fistulas,” Gastrointestinal Endoscopy 65, no. 1 (2007): 70–76, 10.1016/j.gie.2006.04.040. 17185082

[11] H. Jiang , L. Ye , Y. Zhang , et al., “Current Status and Future Trends of Biodegradable Stents for Esophageal Stenosis: A Literature Review,” Chinese Medical Journal 137, no. 21 (2024): 2638–2640, 10.1097/CM9.0000000000003300. 39307925 PMC11556982

[12] A. Godin and M. Liberman , “The Modern Approach to Esophageal Palliative and Emergency Surgery,” Annals of Translational Medicine 9, no. 10 (2021): 905, 10.21037/atm.2020.03.107. 34164539 PMC8184432

[13] H. Wang , M. Ke , W. Li , et al., “Chinese Expert Consensus on Diagnosis and Management of Acquired Respiratory‐Digestive Tract Fistulas,” Thoracic Cancer 9, no. 11 (2018): 1544–1555, 10.1111/1759-7714.12856. 30221470 PMC6209773

[14] T. W. Rice , D. T. Patil , and E. H. Blackstone , “8th Edition AJCC/UICC Staging of Cancers of the Esophagus and Esophagogastric Junction: Application to Clinical Practice,” Annals of Cardiothoracic Surgery 6, no. 2 (2017): 119–130, 10.21037/acs.2017.03.14. 28447000 PMC5387145

[15] J. A. Abelson , J. D. Murphy , B. W. Loo, Jr. , et al., “Esophageal Tolerance to High‐Dose Stereotactic Ablative Radiotherapy,” Diseases of the Esophagus 25, no. 7 (2012): 623–629, 10.1111/j.1442-2050.2011.01295.x. 22168251

[16] N. Martini , J. T. Goodner , G. J. D'Angio , and E. J. Beattie, Jr. , “Tracheoesophageal Fistula due to Cancer,” Journal of Thoracic and Cardiovascular Surgery 59, no. 3 (1970): 319–324. 4190046

[17] A. Balazs , P. K. Kupcsulik , and Z. Galambos , “Esophagorespiratory Fistulas of Tumorous Origin. Non‐Operative Management of 264 Cases in a 20‐Year Period,” European Journal of Cardio‐Thoracic Surgery 34, no. 5 (2008): 1103–1107, 10.1016/j.ejcts.2008.06.025. 18678504

[18] J. Cao , H. Xu , W. Li , et al., “Nutritional Assessment and Risk Factors Associated to Malnutrition in Patients With Esophageal Cancer,” Current Problems in Cancer 45, no. 1 (2021): 100638, 10.1016/j.currproblcancer.2020.100638. 32829957

[19] J. Teerakanok , J. P. DeWitt , E. Juarez , K. Z. Thein , and I. Warraich , “Primary Esophageal Diffuse Large B Cell Lymphoma Presenting With Tracheoesophageal Fistula: A Rare Case and Review,” World Journal of Gastrointestinal Oncology 9, no. 10 (2017): 431–435, 10.4251/wjgo.v9.i10.431. 29085570 PMC5648987

[20] K. E. Koch , A. P. Dhanasopon , and G. A. Woodard , “Airway Esophageal Fistula,” Thoracic Surgery Clinics 34, no. 4 (2024): 405–414, 10.1016/j.thorsurg.2024.07.005. 39332865

[21] Q. Wang , Z. Duan , S. Liu , and R. Shi , “Efficacy and Risk Factors of Stent Placement in the Treatment of Malignant Tracheoesophageal Fistula,” Frontiers in Oncology 6, no. 14 (2024): 1421020, 10.3389/fonc.2024.1421020. PMC11333233 39165687

[22] F. Petrella , L. Spaggiari , F. Acocella , et al., “Airway Fistula Closure After Stem‐Cell Infusion,” New England Journal of Medicine 372, no. 1 (2015): 96–97, 10.1056/NEJMc1411374. 25551543

[23] A. Repici , P. Presbitero , A. Carlino , et al., “First Human Case of Esophagus‐Tracheal Fistula Closure by Using a Cardiac Septal Occluder (With Video),” Gastrointestinal Endoscopy 71, no. 4 (2010): 867–869, 10.1016/j.gie.2009.08.036. 20185124

[24] O. Fruchter , B. A. El Raouf , N. Abdel‐Rahman , et al., “Efficacy of Bronchoscopic Closure of a Bronchopleural Fistula With Amplatzer Devices: Long‐Term Follow‐Up,” Respiration 87 (2014): 227–233, 10.1159/000357074. 24434610

[25] F. J. Herth , S. Peter , F. Baty , R. Eberhardt , J. D. Leuppi , and P. N. Chhajed , “Combined Airway and Oesophageal Stenting in Malignant Airway‐Oesophageal Fistulas: A Prospective Study,” European Respiratory Journal 36, no. 6 (2010): 1370–1374, 10.1183/09031936.00049809. 20525708

